# Association of FAS and FAS Ligand Genes Polymorphism and Risk of Systemic Lupus Erythematosus

**DOI:** 10.1155/2013/176741

**Published:** 2013-11-14

**Authors:** Bita Moudi, Saeedeh Salimi, Farzaneh Farajian Mashhadi, Mahnaz Sandoughi, Zahra Zakeri

**Affiliations:** ^1^Cellular and Molecular Research Center, Zahedan University of Medical Sciences, Zahedan 9816743175, Iran; ^2^Department of Clinical Biochemistry, School of Medicine, Zahedan University of Medical Sciences, Zahedan 9816743175, Iran; ^3^Department of Pharmacology, School of Medicine, Zahedan University of Medical Sciences, Zahedan 9816743175, Iran; ^4^Department of Internal Medicine, School of Medicine, Zahedan University of Medical Sciences, Zahedan 9816743175, Iran

## Abstract

FAS/FASL pathway plays a critical role in maintaining peripheral immune tolerance; therefore, the apoptosis genes, Fas and Fas ligand (FasL), could be suitable candidate genes in human SLE susceptibility. *Materials and Methods*. In this case-control study, 106 SLE patients and 149 sex, age, and ethnicity matched healthy controls were genotyped for the Fas A-670G and FasLC-844T polymorphisms by polymerase chain reaction-restriction fragment length polymorphism method (PCR-RFLP). *Results*. The frequency of -670AA genotype was significantly higher in SLE patients than control group and the risk of SLE was 2.1-fold greater in subjects with AA genotype (*P* = 0.03). The frequency of -670A allele was significantly higher in SLE patients than in controls too (58% versus 49%, *P* = 0.03). The -844CC genotype frequency was significantly higher in SLE patients than in healthy controls and the risk of SLE was 2.8-fold greater in these subjects (*P* = 0.01). The C allele frequency was significantly higher in patients than in controls (69% versus 49%, *P* = 0.001). Increased SLE risk was observed in individuals with combined effect of Fas-670AA and FasL-844CC genotypes (*P* = 0.001). *Conclusion*. Fas-670AA and FasL-844CC genotypes were associated with SLE risk, and combined effect of -670AA and -844CC genotypes might increase SLE susceptibility.

## 1. Introduction

Systemic lupus erythematosus (SLE) is a prototype autoimmune disease that is easily confused with many disorders and can affect different organs including skin, heart, lungs, blood vessels, liver, kidneys, joints, and nervous system. This complication is characterized by malregulation of T and B cells causing production of excessive amounts of different autoantibodies and formation of immune complexes against different nuclear antigens [1, 2].

The prevalence of SLE ranges from approximately 40 cases per 100,000 individuals among Northern Europeans to more than 200 per 100,000 persons among blacks [3]. This disease occurs in women ten times more frequently than in men, especially in women during their reproductive years [4, 5]. Similar to other autoimmune diseases, SLE showed an inheritance pattern, and multiple candidate genes have been studied with SLE susceptibility [4]. 

Several evidence indicates that abnormalities in the apoptosis (programmed cell death) process could be related to development of SLE, and the elimination of autoreactive T or B cells is impaired in this disease [6, 7]. Delayed clearance of apoptotic cells by phagocytes was observed in SLE patients [8]. Also accelerated apoptosis of circulating cells was observed in SLE patients which causes the specific lupus autoantigens, for instance, double stranded DNA (dsDNA), to be exposed on surface blebs [6, 9].

The importance of apoptosis in immune tolerance was distinguished on a study of the genetic defects in FAS and its ligand (FASL) in mouse models of human SLE [10].

FAS/FAS ligand system is the main extrinsic pathway for the initiation of apoptosis in numerous cells and tissues [11]. FAS is a membrane protein of the transmembrane tumor necrosis factor superfamily of death receptors and plays an important role in apoptotic signaling in different cells. This receptor induces apoptosis by binding to its natural ligand. FASL is a member of tumor necrosis factor superfamily and initiates the death signal cascade, which ultimately results in apoptotic cell death. Although FAS is existing constitutively on the surface of resting cells in low levels, it is expressed prominently on the surface of activated T cells [12]. 

FAS and FASL genes are located on chromosomes 10q24.1 and 1q23, respectively [13, 14]. Several polymorphisms have been recognized in FAS gene, and G to A replacement at nucleotide position -1377 (FAS G-1377A, rs2234767) in the silencer region as well as A to G replacement at nucleotide position -670 (FAS A-670G, rs1800682) in the enhancer region are studied more than others [15]. The FASL gene C-844T polymorphism is located in the binding site of transcription factor CAAT/enhancer-binding protein *β*. A markedly elevated basal expression of FASL is observed in subjects with the C allele compared with the T allele at nucleotide position -844 (FASL C-844T, rs763110) in the promoter region [16].

Several studies investigated the role of FAS and FASL gene polymorphisms in the etiology of SLE; however, the role of FAS and FASL gene polymorphisms in lupus has not been conclusively established [16–18]. Therefore, the present study was designed to investigate the association of apoptosis-related genes, FAS and FASL polymorphisms, with SLE susceptibility and clinical characteristics in Iran.

## 2. Materials and Methods

### 2.1. Characteristics of Study Populations

Approval of the study was obtained from the Ethics Committee of Zahedan University of Medical Sciences. This case-control study, performed on 106 SLE patients (9 men and 97 women) with a mean age of 31.8 ± 7.8 years, who fulfilled the 1998 American College of Rheumatology (ACR) criteria, was referred to rheumatology clinics of Ali-Ebne-Abitaleb hospital in Zahedan from 2011 to 2013. The control group consists of 149 age, sex, and ethnically matched volunteers (13 men and 139 women) with a mean age of 32.90 ± 13.3 years. The healthy controls were selected following a questionnaire survey to exclude any systemic, inflammatory, and autoimmune diseases with negative ANA test and no family relation with lupus patients. A written informed consent was obtained from all participants.

### 2.2. Genotyping

Genomic DNA was extracted from ethylene diamine tetra-acetic acid (EDTA) anticoagulated peripheral blood by DNA isolation kit (Roche, Germany). FAS and FASL polymorphisms were identified by the PCR-RFLP method.

The primer sequences, annealing temperature, restriction enzymes, and fragments size are shown in Table 1. 

Polymerase chain reaction was performed in thermal cycler (Eppendorf-Mastercycler Gradient-Germany) in a 25 *μ*L final volume which contained 20 pmol of each primer, 0.1 mmol of dNTP (Fermentas, Lithuania), 0.3 *μ*g of genomic DNA, 1.5 mmol/L of MgCl_2_, 2.5 *μ*L of 10X PCR buffer, and 1.5 unit of Taq DNA polymerase (Fermentas, Lithuania), according to the following protocol: initial denaturation at 95°C for 5 min; 30 cycles of denaturation at 95°C for 1 min, annealing for 1 min at 61°C for FAS A-670G and 63°C for FASL C-844T polymorphisms; extension at 72°C for 2 min; and final extension at 72°C for 5 minutes. PCR and digested products were separated by electrophoresis on a 2% agarose gel and visualized by ethidium bromide staining.

### 2.3. Statistical Analyses

The statistical analysis of the data was performed using the SPSS 15.0 software. Quantitative data were presented as mean ± standard deviation. Genotypes and alleles were compared between groups by use of *χ* ^2^ test. Comparison of quantitative variants between two groups was assessed by Student's *t*-test. The association between qualitative variables was analyzed using the chi-square or Fisher exact tests. Variations with *P* values less than 0.05 were considered significant. Odds ratio and 95% confidence interval were calculated to evaluate the strength of the associations. 

## 3. Results

Clinical and demographic characteristics of SLE patients and control samples were shown in Table 2. The SLE patients and controls did not differ significantly with respect to sex, age, and ethnic parameters. Skin manifestations, arthritis, lupus nephritis, and neuropsychiatric manifestations developed in 83%, 87%, 22%, and 14% of patients, respectively, during the course of their disease.

Allele and genotype frequencies of both polymorphisms were in Hardy-Weinberg equilibrium in case and control groups. The frequency of the AA genotype of FAS A-670G polymorphism was significantly higher in SLE patients than in controls, and the risk of SLE was 2.1-fold greater in individuals with AA genotype in comparison to GG genotype (OR, 2.1 (95% CI, 1 to 4.1); *P* = 0.03). The frequency of A allele was statistically higher in SLE group (58%) than in controls (49%) too (OR, 1.4 (95% CI, 1 to 2); *P* = 0.03). 

Furthermore, the frequency of CC genotype of C-844T polymorphism of FASL gene was significantly higher in SLE patients than in healthy controls, and the risk of SLE was 2.8-fold higher in individuals with CC genotype in comparison to TT genotype (OR, 2.8 (95% CI, 1.2 to 6.2); *P* = 0.01). The frequency of C allele was 69 and 54 percent in SLE and control groups, respectively, and was significantly different (OR, 1.9 (95% CI, 1.3 to 2.7); *P* = 0.001) (Table 3).

Analysis of the synergic effects of various genotypes on risk of SLE showed that the individuals with the AA/TT genotype for FASA-670G and FASLC-844T polymorphisms showed 3.7-fold increase in SLE risk compared to GG/TT genotype (OR, 3.7 (95% CI, 1.1 to 12.5); *P* = 0.03). Also 5.6-fold increased risk of SLE was found by comparing AA/TT genotype with GG/TT, GG/CT, and AG/TT genotypes between the case and control groups (OR, 5.6 (95% CI, 1.9 to 15.6); *P* = 0.001) (Table 4). 

Moreover, no minor alleles frequency differences were observed between two ethnic groups and different manifestations.

## 4. Discussion

Established evidence shows that failure of apoptosis, or programmed cell death, is considered to contribute to the development of autoimmune disorders characterized by the impaired elimination of autoreactive T cells or B cells [6, 7]. Moreover, it appears that increase of lymphocyte apoptosis and deficient phagocyte-mediated clearance of apoptotic cells could contribute to B-cell hyperactivity and subsequent autoantibodies overproduction [8]. Apoptotic cells are the major source of autoantigens production in SLE pathogenesis. The autoantigens trigger autoantibody production and subsequently induce systemic autoimmunity, leading to formation of many apoptotic blebs [8, 19, 20]. The accelerated apoptosis may be a direct consequence of alterations in proteins/genes related to programmed cell death, such as FAS and FASL [21]. 

Although the role of FAS-mediated apoptosis in immunity and elimination of autoreactive lymphocytes are clear, but the function of FAS or FASL as the markers of apoptosis in autoimmune disorders, especially SLE, needs more investigations [22]. Nevertheless, several studies indicated that FAS may be involved in the defective apoptosis of T cells in SLE, and resistance to the FAS-mediated apoptosis has been observed in T cells from SLE patients [23, 24].

The FAS/FASL system stimulates immune tolerance by the apoptosis induction and elimination of activated T, B lymphocytes and macrophages [24]. 

Mouse model investigations indicated that FAS or FASL mutations could initiate autoimmune diseases. The essential role could be played by the FAS/FASL system in the maintenance of immune tolerance, and inhibition of autoimmune disease has been clearly established [25]. 

In addition abnormal expression of FAS and FASL on T and B lymphocytes in SLE patients has been demonstrated in several reports [26, 27]. 

The FAS gene has a A to G substitution in the promoter region at position -670, which decreases FAS production [15]. Also higher basal expression of FASL gene is observed in association with the C allele compared with the T allele of C-844T polymorphism of FASL [16]. 

Since these two polymorphisms could alter basal expression of FAS and FASL genes, recent studies have suggested that FAS and FASL gene polymorphisms may play important roles in pathogenesis of SLE.

In the present study, a significant difference in AA genotype frequency was observed between SLE patients and controls in comparison to GG genotype, and the risk of SLE was 2.1-fold higher in subjects with AA genotype. Also the frequency of A allele was significantly higher than G allele in SLE patients than in healthy controls. 

For the first time, Wu et al. in 1996 reported an association between a 84 bp deletion within exon 4 (28 amino acid deletion) of the FASL gene and SLE in USA. This deletion in extracellular domain of the FASL gene was shown to cause defect in cell death and FASL-mediated apoptosis [28]. Furthermore, in 1996, Huang et al. screened the whole 5′ flanking region of the FAS gene and identified the A-670G and G-1377A polymorphisms within the silencer and enhancer regions of this gene, respectively [29]. Then in 1999, the association between FAS A-670G polymorphism and SLE was investigated in Australians, and higher frequency of GG genotype in SLE patients with photosensitivity or oral ulcers was found [18]. 

An association between FAS A-670G polymorphism and development of anti-RNP antibodies was observed by Lee et al. in SLE patients [30]. 

In consistent with present study, Kanemitsu reported that the A allele of FAS A-670G but not G-1377A was significantly associated with SLE [31].

In contrast to present study Araste et al. reported no different allelic distributions at position -670 between patients and controls in an Iranian population. But they observed higher GG genotype and G allele distribution at position -1377 in SLE patients. Also they found higher soluble FAS and FAS ligand levels in the patient group and lower amounts of serum anti-SSB/La in patients with the -670GG genotype [32].

Our data supported two separate meta-analysis results that performed recently by Lee et al. in 2012 and Xiang et al. in 2013 [17, 33]. Lee et al. carried out a meta-analysis to search the effect of FAS A-670G polymorphism on susceptibility to autoimmune rheumatic diseases. They revealed an association between rheumatic diseases and the FAS A-670G polymorphism in the dominant model. Moreover, they indicated an association between the FAS-670 G allele carrier and rheumatic diseases in the Asian. They observed a negative association between the FAS-670 G allele and SLE susceptibility (OR = 0.578, 95% CI = 0.358–0.934, *P* = 0.025). In another meta-analysis that conducted by Xiang et al., similar results were achieved [17, 33].

Moreover, we revealed that CC genotype of FASL C-844T polymorphism was significantly higher in SLE patients than in healthy controls, and the frequency of C allele was statistically higher in SLE patients.

 Although several studies are performed to investigate the effect of FASL gene mutations on SLE susceptibility, to date there are only a few published reports about the association between C-844T polymorphism and SLE [16, 33]. 

In 2003, Wu et al. sequenced the coding region and proximal 1 kb of promoter region of FASL gene in 14 SLE patients, 30 rheumatoid arthritis patients, and 7 normal subjects. They found an SNP at -844 within the FASL promoter region (T allele in addition to the C) in normal populations with an allele frequency of 0.82 for African Americans. Also they reported increased expression of FASL in individuals with -844CC genotype that could enhance autoimmunity risk. In this study a significant association between -844CC genotype and SLE was reported in the African Americans in comparison to ethnically matched healthy controls. Although they showed higher frequency of -844CC genotype in Caucasian SLE patients, the difference was not statistically significant [16].

Similarly, in 2005, Chen et al. revealed that -844CC genotype and C allele were associated with lupus susceptibility; however, they found that the -1094A/C polymorphism is not associated with SLE [33]. 

As mentioned above our findings are in line with those reported by Wu et al. and Chen et al. results and supported the association of FAS ligand C-844T polymorphism and SLE hypothesis.

Since the effect of a single SNP is generally minor, it is believed that the combination effects of functionally relevant SNPs may contribute to increased SLE risk. So, we studied the combined polymorphism of FAS and FASL genes and SLE susceptibility.

Our results revealed that the combination of AA/CC genotypes of FAS A-670G/FASL C-844T polymorphisms might increase SLE risk approximately 3.7-fold compared to GG/CC genotype. Also 5.6-fold increased SLE risk was observed in individuals with AA/CC genotype in comparison with GG/TT, GG/CT, and AG/TT genotypes. To the best of our knowledge, the present study is the first investigation about the synergic effect of FAS A-670G and FAS ligand C-844T polymorphisms on SLE susceptibility. These results need more study to explore gene-gene interactions and its relation to SLE susceptibility to identify individuals at increased risk of SLE and to develop preventive strategies.

There were not any differences in minor allele frequencies between two ethnic groups and various SLE manifestations.

In conclusion, we found an association between AA genotype of FAS A-670G polymorphism and CC genotype of FASL C-844T polymorphism with SLE risk. Also the combination of AA/CC genotypes of FAS A-670G and FASL C-844T polymorphisms indicated higher SLE risk in this Iranian population.

## Figures and Tables

**Table 1:** The primer sequences, annealing temperature, restriction enzymes, and fragments size of FAS and FASL polymorphisms.

Target sequence	Primer sequence	Annealing temperature	Restriction enzyme	PCR product	RFLP fragments
FAS A-670G rs1800682	Forward: 5′-CTACCTAAGAGCTATCTACCGTTC-3′ Reverse: 5′-GGCTGTCCATGTTGTGGCTGC-3′[29]	61°C	MvaI (BstNI)	223	A: 223 G: 44, 189
FASL C-844T rs763110	Forward: 5′-CAGCTACTCGGAGGCCAAG-3′ Reverse: 5′-GCTCTGAGGGGAGAGACCAT-3′[19]	63°C	BseMI (BsrDI)	401	T: 401 C: 223, 168

**Table 2:** Demographic characteristics of SLE patients and controls.

Parameter	SLE *N* = 106	Controls *N* = 103	*P *value	*x* ^2^
Age (year)	31.8 ± 7.8	32.90 ± 13.3	0.53	0.04
Sex (male/female)	9.97	8.95	0.5	0.04
Race *N* (%)				
Persian	49 (46)	53 (51)	0.27	0.27
Balouch	57 (54)	50 (49)		

**Table 3:** Genotype and allele frequencies of FAS A-670G and FASL C-844T polymorphisms in SLE patients and controls.

	SLE *N* = 106	Control *N* = 149	*P *value	Odds ratio (95% CI)
FAS A-670G				
AA, *n* (%)	34 (32)	37 (25)	0.03	2.1 (1–4.1)
AG, *n* (%)	55 (52)	73 (49)	0.3	1.2 (0.7–2.2)
GG, *n* (%)	17 (16)	39 (26)		Ref = 1
A *n* (%)	123 (58)	147 (49)	0.03	1.4 (1-2)
G *n* (%)	89 (42)	151 (51)		Ref = 1
FAS ligand C-844T				
CC, *n* (%)	50 (47.2)	51 (34.2)	0.01	2.8 (1.2–6.2)
CT, *n* (%)	46 (43.4)	80 (47)	0.1	1.8 (0.8–4.2)
TT, *n* (%)	10 (9.4)	28 (18.8)		Ref = 1
C *n* (%)	146 (69)	162 (54)	0.001	1.9 (1.3–2.7)
T *n* (%)	66 (31)	136 (46)		Ref = 1

**Table 4:** Synergic effect of FAS A-670G and FAS ligand C-844T polymorphisms in SLE patients and controls.

FAS A-670G/FASL C-844T	SLE *N* = 106	Control *N* = 149	*P *value	OR (95% CI)
**GG/TT**	**7 (6.6)**	**13 (9.9)**		**Ref = 1**
AG/CT	29 (27.4)	37 (25.9)	0.3	1.4 (0.5–4.2)
AG/CC	25 (23.6)	30 (21)	0.3	1.4 (0.5–4.6)
AA/TT	2 (1.9)	8 (5.6)	0.3	0.45 (0.08–2.9)
AA/CT	14 (13.2)	18 (12.6)	0.4	1.5 (0.5–5)
AA/CC	18 (17)	9 (6.3)	0.03	3.7 (1.1–12.5)
**GG/TT, GG/CT, AG/TT**	**11 (10.4)**	**30 (20)**		**Ref = 1**
AG/CT	29 (27.4)	37 (25.9)	0.06	2.1 (0.9–5)
AG/CC	25 (23.6)	30 (21)	0.05	2.3 (0.9–5.4)
AA/TT	2 (1.9)	8 (5.6)	0.5	0.67 (0.13–3.3)
AA/CT	14 (13.2)	18 (12.6)	0.5	2.1 (0.8–5)
AA/CC	18 (17)	9 (6.3)	0.001	5.6 (1.9–15.6)

## References

[1] Rahman A, Iseberg DA (2008). Mechanisms of disease. Systemic lupus erythematosus. *The New England Journal of Medicine*.

[2] Kotzin BL (1996). Systemic lupus erythematosus. *Cell*.

[3] Johnson AE, Gordon C, Palmer RG, Bacon PA (1995). The prevalence and incidence of systemic lupus erythematosus in Birmingham, England: relationship to ethnicity and country of birth. *Arthritis and Rheumatism*.

[4] Nath SK, Kilpatrick J, Harley JB (2004). Genetics of human systemic lupus erythematosus: the emerging picture. *Current Opinion in Immunology*.

[5] Rus VHA, Hochberg MC, Silman AJHM (2001). Systemic lupus erythematosus. *Epidemiology of the Rheumatic Diseases*.

[6] Andrade F, Casciola-Rosen L, Rosen A (2000). Apoptosis in systemic lupus erythematosus: clinical implications. *Rheumatic Disease Clinics of North America*.

[7] Munoz LE, Van Bavel C, Franz S, Berden J, Herrmann M, Van Der Vlag J (2008). Apoptosis in the pathogenesis of systemic lupus erythematosus. *Lupus*.

[8] Ren Y, Tang J, Mok MY, Chan AWK, Wu A, Lau CS (2003). Increased apoptotic neutrophils and macrophages and impaired macrophage phagocytic clearance of apoptotic neutrophils in systemic lupus erythematosus. *Arthritis and Rheumatism*.

[9] Green DR (2003). Overview: apoptotic signaling pathways in the immune system. *Immunological Reviews*.

[10] Nagata S, Suda T (1995). Fas and Fas ligand: lpr and gld mutations. *Immunology Today*.

[11] Nagata S (1994). Fas and Fas ligand: a death factor and its receptor. *Advances in Immunology*.

[12] Kamradt T, Mitchison NA (2001). Tolerance and autoimmunity. *The New England Journal of Medicine*.

[13] Behrmann I, Walczak H, Krammer PH (1994). Structure of the human APO-1 gene. *European Journal of Immunology*.

[14] Takahashi T, Tanaka M, Inazawa J, Abe T, Suda T, Nagata S (1994). Human Fas ligand: gene structure, chromosomal location and species specificity. *International Immunology*.

[15] Pinti M, Troiano L, Nasi M (2002). Genetic polymorphisms of Fas (CD95) and FasL (CD178) in human longevity: studies on centenarians. *Cell Death and Differentiation*.

[16] Wu J, Metz C, Xu X (2003). A novel polymorphic CAAT/enhancer-binding protein *β* element in the FasL gene promoter alters Fas ligand expression: a candidate background gene in African American systemic lupus erythematosus patients. *Journal of Immunology*.

[17] Xiang N, Li XM, Wang GS, Tao JH, Li XP (2013). Association of FAS gene polymorphisms with systemic lupus erythematosus: a meta-analysis. *Molecular Biology Reports*.

[18] Huang QR, Danis V, Lassere M, Edmonds J, Manolios N (1999). Evaluation of a new Apo-1/Fas promoter polymorphism in rheumatoid arthritis and systemic lupus erythematosus patients. *Rheumatology*.

[19] Sun T, Miao X, Zhang X, Tan W, Xiong P, Lin D (2004). Polymorphisms of death pathway genes FAS and FASL in esophageal squamous-cell carcinoma. *Journal of the National Cancer Institute*.

[20] Courtney PA, Crockard AD, Williamson K, Irvine AE, Kennedy RJ, Bell AL (1999). Increased apoptotic peripheral blood neutrophils in systemic lupus erythematosus: relations with disease activity, antibodies to double stranded DNA, and neutropenia. *Annals of the Rheumatic Diseases*.

[21] Liphaus BL, Kiss MHB (2010). The role of apoptosis proteins and complement components in the etiopathogenesis of systemic lupus erythematosus. *Clinics*.

[22] De Maria R, Testi R (1998). Fas-FasL interactions: a common pathogenetic mechanism in organ-specific autoimmunity. *Immunology Today*.

[23] Budagyan VM, Bulanova EG, Sharova NI, Nikonova MF, Stanislav ML, Yarylin AA (1998). The resistance of activated T-cells from SLE patients to apoptosis induced by human thymic stromal cells. *Immunology Letters*.

[24] Fukuyama H, Adachi M, Suematsu S (2002). Requirement of FAS expression in B cells for tolerance induction. *European Journal of Immunology*.

[25] Takahashi T, Tanaka M, Brannan CI (1994). Generalized lymphoproliferative disease in mice, caused by a point mutation in the Fas ligand. *Cell*.

[26] Kovacs B, Liossis S-NC, Dennis GJ, Tsokos GC (1997). Increased expression of functional Fas-ligand in activated T cells from patients with systemic lupus erythematosus. *Autoimmunity*.

[27] Suzuki N, Sakane T (1996). Abnormal Fas and Fas ligand expression of lymphocytes in patients with SLE. *Nippon Rinsho: Japanese Journal of Clinical Medicine*.

[28] Wu J, Wilson J, He J, Xiang L, Schur PH, Mountz JD (1996). Fas ligand mutation in a patient with systemic lupus erythematosus and lymphoproliferative disease. *Journal of Clinical Investigation*.

[29] Huang QR, Morris D, Manolios N (1997). Identification and characterisation of polymorphisms in the promoter region of the human Apo-1/Fas (CD95) gene. *Molecular Immunology*.

[30] Lee YH, Kim YR, Ji JD, Sohn J, Song GG (2001). Fas promoter -670 polymorphism is associated with development of anti-RNP antibodies in systemic lupus erythematosus. *Journal of Rheumatology*.

[31] Kanemitsu S, Ihara K, Saifddin A (2002). A functional polymorphism in Fas (CD95/APO-1) gene promoter associated with systemic lupus erythematosus. *Journal of Rheumatology*.

[32] Araste JM, Sarvestani EK, Aflaki E, Amirghofran Z (2010). Fas gene polymorphisms in systemic lupus erythematosus and serum levels of some apoptosis-related molecules. *Immunological Investigations*.

[33] Chen J-Y, Wang C-M, Ma C-C, Chow Y-H, Luo S-F (2005). The −844C/T polymorphism in the Fas ligand promoter associates with Taiwanese SLE. *Genes and Immunity*.

